# The impact of coronary outflow and non-Newtonian fluid property on aortic valve haemodynamics

**DOI:** 10.1007/s10237-025-01975-2

**Published:** 2025-06-13

**Authors:** Zhongjie Yin, Chlöe Armour, Harkamaljot Kandail, Declan P. O’Regan, Toufan Bahrami, Saeed Mirsadraee, Selene Pirola, Xiao Yun Xu

**Affiliations:** 1 Department of Chemical Engineering, Imperial College London, South Kensington Campus, London, UK; 2 National Heart and Lung Institute, Imperial College London, London, UK; 3 Medtronic Neurovascular, Irvine, CA USA; 4 Laboratory of Medical Sciences, Imperial College London, London, UK; 5 Department of Cardiothoracic Surgery, Royal Brompton and Harefield Hospitals NHS Trust, London, UK; 6 Department of Radiology, Royal Brompton and Harefield Hospitals NHS Trust, London, UK; 7 Department of BioMechanical Engineering, TU Delft, Delft, The Netherlands

**Keywords:** Aortic valve, Fluid–structure interaction, Coronary outflow, Non-Newtonian blood property, Wall shear stress

## Abstract

The normal healthy aortic valve (AoV) has three leaflets, two of which have outflows to the coronary arteries. Blood flow through the coronary ostia will have an impact on AoV dynamics and the surrounding haemodynamics, leading to differential shear stress distributions at the aortic side of the three leaflets. In addition, aortic root haemodynamics may also be influenced by the non-Newtonian behaviour of blood which is known as a shear-thinning fluid due to the aggregation of red blood cells at low shear rate. However, the combined effect of coronary and non-Newtonian flow on AoV haemodynamics has not been studied in an anatomically realistic setting. In this study, strongly coupled fluid–structure interaction (FSI) analyses were performed on a natural, healthy AoV, with and without accounting for coronary outflows and non-Newtonian properties of blood. Our results showed that the influence of coronary outflow is more pronounced than employing a non-Newtonian model, and their combined effect is non-negligible, particularly on wall shear stress. Incorporating coronary outflow and non-Newtonian properties increased time-averaged wall shear stress (TAWSS) in the aortic sinus by up to 108.45%; it also increased TAWSS on the aortic side of valve leaflets by 41.04%, 44.76%, and 54.91% on the left, right and non-coronary leaflet, respectively. These results highlight the importance of incorporating coronary outflow and non-Newtonian properties when accurate predictions of wall shear stress and its related parameters are critical.

## Introduction

A normal healthy aortic valve (AoV) consists of three leaflets located in the left, right and posterior directions of the heart. Two of these leaflets have outflows to the coronary arteries and are known as the left coronary leaflet (LCL) and right coronary leaflet (RCL). The third leaflet is known as the non-coronary leaflet. As a result of this anatomical configuration, blood flow through the coronary ostia will have an impact on flow patterns in the aortic root and shear stress distributions on the aortic wall and valve leaflets.

Previous clinical studies have shown that coronary artery diseases are a potential risk for AoV replacement (Faroux et al. 2019; Mullany et al. 1987; Wiegerinck et al. 2015). Several experimental (Gaillard et al. 2010; Nakao et al. 1987) and computational studies (Kivi et al. 2020; Mohammadi et al. 2017; Wald et al. 2018) also indicated that impairment in AoV could lead to abnormal coronary haemodynamics, such as variations in blood velocity and wall shear stress (WSS). However, most of these studies focused on the impact of AoV on coronary flow, while only a few examined the influence of coronary flow on the function of AoV and its surrounding haemodynamics. Moore et al. (2015) showed how coronary flow changed the sinus haemodynamics, fluid shear stress and valve dynamics using an experimental method. Cao et al. (2016, 2017) revealed that including coronary flow in fluid–structure interaction (FSI) simulations of the aortic root increased WSS on all three leaflets and caused the magnitude and direction of WSS to differ among the three leaflets, but they employed an idealized aortic root model and symmetric valve geometry. Although coronary arteries have been included in several AoV FSI studies (Kandail et al. 2018; Kivi et al. 2020; Emendi et al. 2021), the impact of coronary flow on valve dynamics and shear stress on leaflet surfaces has not been quantitatively analysed in anatomically realistic aortic root geometry.

Blood is known as a shear-thinning fluid due to the aggregation of red blood cells in low shear rate conditions (Murata 1976), and the non-Newtonian effect of blood on flow in the aorta has been studied extensively (Qiao et al. 2024). However, most AoV-related FSI studies treated blood as a homogeneous Newtonian fluid; only a few FSI simulations of mechanical valves employed a non-Newtonian model (Chen et al. 2022; De Vita et al. 2015). Although shear rate in large arteries is generally high (Fung 2013), there are localized regions of flow stagnation or disturbed flow where shear rate is low, and one such example is the aortic sinuses (Ghasemi Pour et al. 2022; Mutlu et al. 2021; Tango et al. 2018). Therefore, it is also important to assess the impact of non-Newtonian viscous behaviour on FSI simulations of AoV.

This study focuses on examining the individual and combined effects of coronary outflow and non-Newtonian fluid properties on AoV dynamics and the surrounding haemodynamics by performing strongly coupled FSI analyses on a natural, healthy AoV under subject-specific flow conditions. Qualitative and quantitative comparisons were made in terms of flow pattern, valve dynamics and WSS-derived metrics for different model combinations. Predicted key haemodynamic parameters were also verified against in vivo measurements made with 4D-flow magnetic resonance imaging (MRI).

## Methods

### Model geometry and simulation setup

**Fig. 1:**
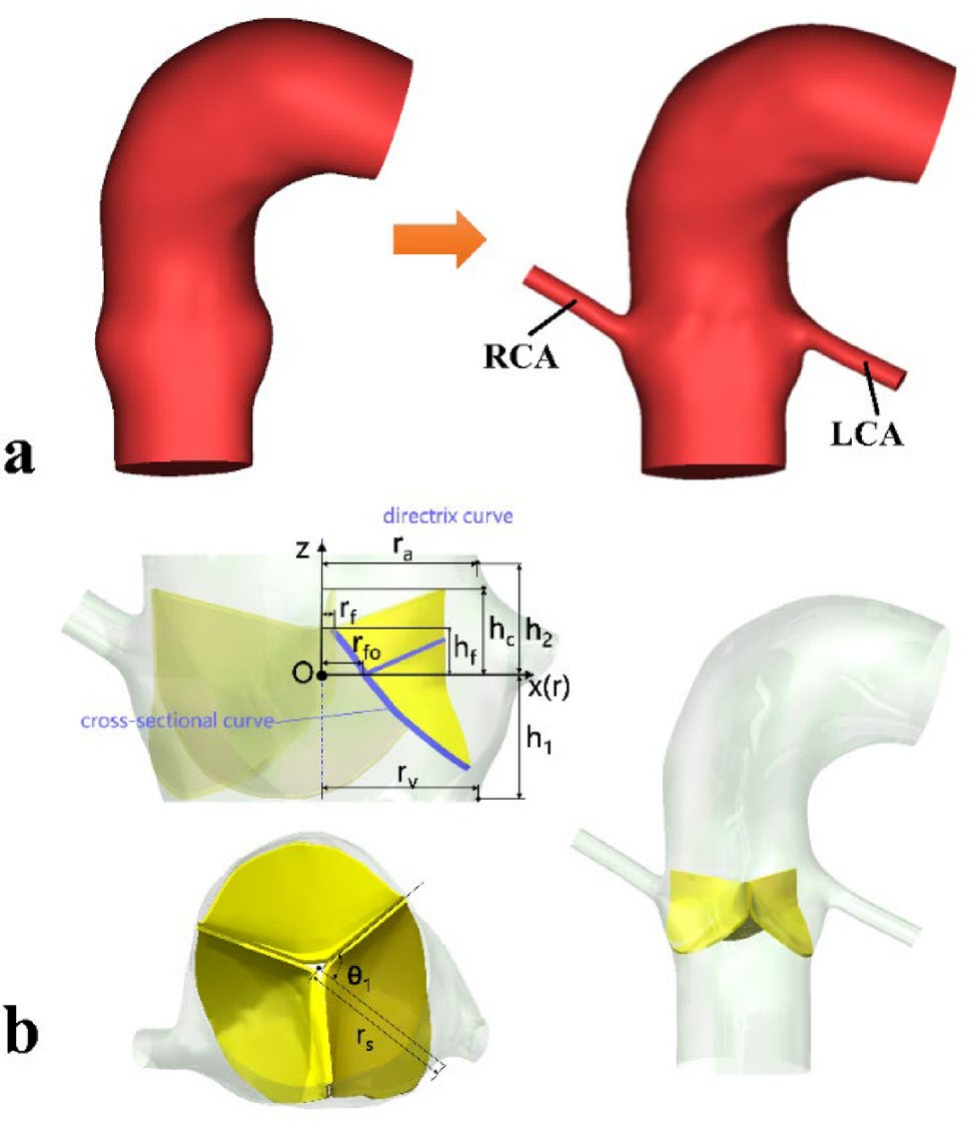
Geometric models of the aortic root and valve leaflets: **a** subject-specific models without (left) and with (right) the left and right coronary arteries (LCA and RCA); **b** parametric valve leaflets and the integrated model

The geometric model of the aortic root and valve leaflets reported in our previous publication (Yin et al. 2024) was adopted. To account for the effect of coronary outflow, the aortic root model was modified to include the left coronary artery (LCA) and right coronary artery (RCA) by reconstructing them from the same set of MR image data via Materialise Mimics (v24.0, Materialise, Leuven, Belgium). Figure 1 shows the geometric models with and without the coronary arteries, and the integrated AoV leaflets based on the parametric model proposed by Haj-Ali et al. (2012). The key parameters of this model are summarized in Table 1. AoV leaflets were discretized with C3D8R hexahedral elements in Abaqus (SIMULIA, Dassault System, France). A global solid mesh size of 0.25 mm and four elements in the thickness direction were found to be sufficient following a solid mesh sensitivity analysis performed in our previous study (Yin et al. 2024). The material of the AoV leaflets was described using an isotropic, hyperelastic, third-order Ogden model (1972), and all material parameters had the same values as in our previous study (Yin et al. 2024).

**Table 1:** Values of key parameters (defined in Fig. 1b) for the three leaflets

Parameter	Description	NCL	LCL	RCL
*r*_*v*_	Sinus radius (ventricular end)	13.52 mm	12.63 mm	12.99 mm
*r*_*a*_	Sinus radius (aortic end)	13.55 mm	12.02 mm	12.97 mm
*r*_*s*_	Sinus radius (z = 0 plane)	14 mm	15.35 mm	15.58 mm
*θ*_*1*_	Angle between the centreline of leaflet and neighbouring leaflet	60°	62.5°	57.5°
*h*_*1*_	Distance between the ventricular end and z = 0 plane	7 mm		
*h*_*2*_	Distance between the aortic end and z = 0 plane	4*h*_*1*_/3		
*h*_*f*_	Distance between the free edge of leaflets and z = 0 plane	7*h*_*1*_/12		
*h*_*c*_	Distance between the commissure of leaflets and z = 0 plane	5*h*_*1*_/6		
*r*_*fo*_	Leaflet radius (z = 0 plane)	0.27*r*_*v*_		
*r*_*f*_	Leaflet radius (free edge)	0		

Table 2 shows the four FSI simulation cases included in this study. The baseline case, NC-N (non-coronary model with a Newtonian fluid), has already been presented in our previous work (Yin et al. 2024). Case C-N was designed to isolate the effect of coronary outflow, while case NC-NN was designed to determine the non-Newtonian effect. Finally, case C-NN incorporated both coronary outflow and non-Newtonian effect.

**Table 2:** Summary of the four simulation cases. LVOT, left ventricular outflow tract

Case name	Range of aorta geometry	Assumption of fluid properties
NC-N	LVOT to ascending aorta without coronary arteries	Newtonian
C-N	LVOT to ascending aorta with coronary arteries	Newtonian
NC-NN	LVOT to ascending aorta without coronary arteries	Non-Newtonian
C-NN	LVOT to ascending aorta with coronary arteries	Non-Newtonian

In the cases where blood was assumed to be a Newtonian fluid (cases NC-N and C-N), the dynamic viscosity was set to be 0.0035 Pa·s. In the cases where blood was treated as a non-Newtonian fluid (cases NC-NN and C-NN), the Bird–Carreau model was adopted to describe its shear-thinning viscous behaviour, which is written as:

1 $$\mu = \mu_{\infty } + \left( {\mu_{0} - \mu_{\infty } } \right)\left[ {1 + \left( {\lambda \dot{\gamma }} \right)^{2} } \right]^{{\frac{n - 1}{2}}}$$

where $\mu$ is blood viscosity, ${\mu }_{\infty }$ is the high-shear viscosity, ${\mu }_{0}$ is the low-shear viscosity, $n$ is the power law index, $\lambda$ is the time constant, and $\dot{\gamma }$ is the shear rate. Here, the following values were adopted: ${\mu }_{\infty }=0.0035 Pa\bullet s$, ${\mu }_{0}=0.056 Pa\bullet s$, $n=0.3568$ and $\lambda =3.313 s$ (Cho and Kensey 1991). These parameters were adopted following a recent computational study (Wang et al. 2023) where the same parameters were used in their thrombosis predictive model, demonstrating good agreement with in vitro experimental data on thrombus formation in a backward-facing step and in vivo data on false lumen thrombosis in aortic dissection. In all cases, blood was assumed to be an incompressible fluid with a density of 1060 kg/m^3^. To avoid introducing additional complexities in the FSI simulation, the flow was assumed to be laminar with the aortic wall being rigid.

### Boundary conditions and computational details

**Fig. 2:**
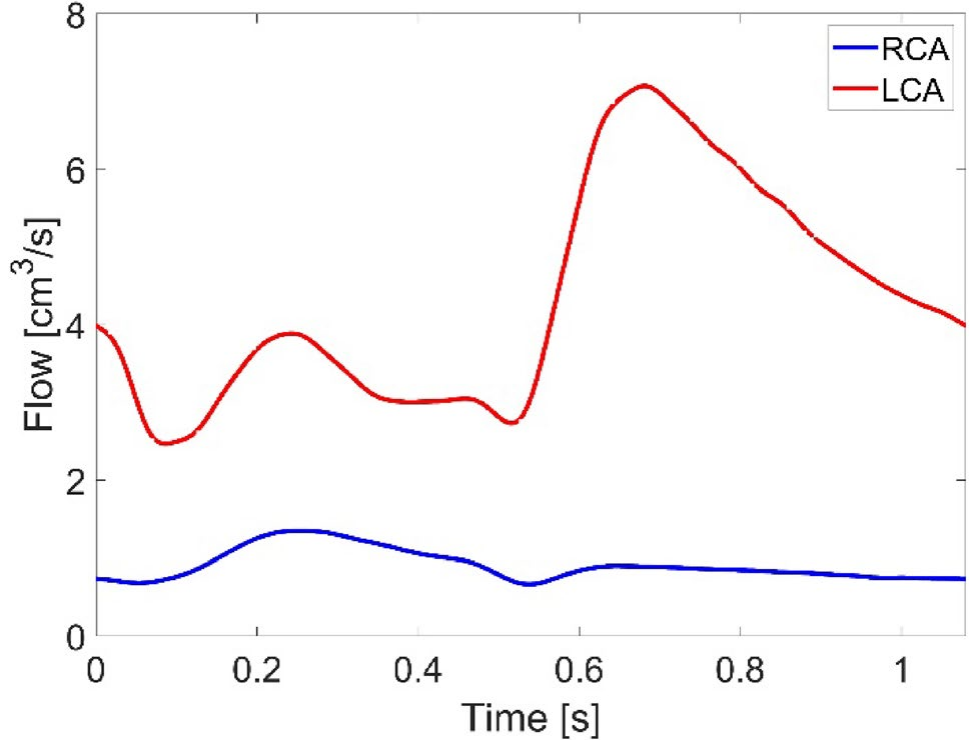
Flow waveform applied at the coronary outlets

Following the procedure described in our previous work (Yin et al. 2024), in the fluid domain, a total pressure waveform was prescribed at the model inlet located in the left ventricular outflow tract (LVOT), and a three-element Windkessel (3EWK) model was applied at the descending aorta outlet. For cases including the coronary arteries (C-N and C-NN), coronary flow waveforms corresponding to a healthy volunteer (Kim et al., 2010) were scaled to fit the current case in two steps. First, the LCA and RCA waveforms were scaled along the time-axis to match the systolic and diastolic phase extracted from the LVOT flow waveform (Yin et al. 2024); the amplitude of the resulting waveforms was then scaled to ensure the time-averaged flow was 3.1% and 0.65% of the LVOT flow for the LCA and RCA (Kandail et al. 2018), respectively. The modified flow waveforms, shown in Fig. 2, were applied at the coronary outlets. Table 3 shows the values of 3EWK parameters adopted for the four simulation cases. There are differences between the models with and without coronary arteries due to outflows through the LCA and RCA. In the structural domain, the attachment edges of all leaflets were fully constrained. The contact model among the three leaflets was defined using a ‘hard’ normal pressure-overclosure formulation, whereas tangential behaviours were defined as a penalty with a friction coefficient of 0.01.

**Table 3:** 3EWK parameters for the descending aorta outlet of four cases (NC-N, C-N, NC-NN, C-NN). ${R}_{p}$: proximal resistance, ${R}_{d}$: distal resistance, $C$: compliance

Case name	${R}_{p}$(kg/m^4^s)	${R}_{d}$(kg/m^4^s)	$C$(m^4^s^2^/kg)
NC-N, NC-NN	1.903 × 10^7^	7.030 × 10^7^	2.004 × 10^–8^
C-N, C-NN	1.829 × 10^7^	7.498 × 10^7^	1.919 × 10^–8^

A two-way FSI coupling was set up between Abaqus Explicit 2019 (SIMULIA, Dassault System, France), served as the solid solver, and FlowVision 3.13.04 (CAPVIDIA, Leuven, Belgium), served as the fluid solver, at an exchange time step of 0.0001 s. At every exchange time step, the fluid solver calculates the concentrated forces based on the resolved velocity and pressure fields and transfers them to the solid solver as a loading condition. Then, the solid solver calculates the valve deformation, and the deformed geometry is sent back to the fluid solver for the next exchange step. An implicit time integration scheme was used in FlowVision with a constant time step equal to the exchange step (0.0001 s), while the minimum explicit time step used in Abaqus was approximately 4 × 10^–7^ s. The fluid mesh for cases C-N and C-NN was built based on cases NC-N and NC-NN, and including coronary arteries added approximately 10,000 additional computational cells. The fluid mesh sensitivity tests were performed to make sure the variables of interest were still in good convergence (< 5% difference in downstream velocity and shear stress on the valve leaflets) after the non-Newtonian assumption was applied and coronary arteries were included (case C-NN). As shown in Table 4, mesh M4 was deemed sufficient for the models with coronary arteries. Three cardiac cycles were run for each FSI simulation to reach a periodic solution, and the difference in LVOT flow rate between the second and the third cycle was less than 5%. Results obtained from the third cycle were used for post-processing and further analysis.

**Table 4:** Fluid mesh sensitivity test results for the aortic root model with coronary arteries and non-Newtonian fluid and a healthy valve (case C-NN). All values are time-averaged over the cardiac cycle

Name	Grid Number	Averaged Velocity DOWN1	Averaged Velocity DOWN2	Averaged Velocity DOWN3	Averaged WSS NCL	Averaged WSS RCL	Averaged WSS LCL
M1	266,264	0.610 m/s	0.511 m/s	0.546 m/s	5.450 Pa	6.320 Pa	6.171 Pa
M2	448,620	0.624 m/s	0.532 m/s	0.558 m/s	6.011 Pa	6.642 Pa	6.561 Pa
M3	613,873	0.639 m/s	0.535 m/s	0.554 m/s	5.960 Pa	7.083 Pa	7.068 Pa
M4	882,723	0.637 m/s	0.535 m/s	0.560 m/s	6.601 Pa	7.128 Pa	7.453 Pa
M5	1,092,394	0.626 m/s	0.532 m/s	0.556 m/s	6.645 Pa	7.213 Pa	7.331 Pa
M6	1,381,506	0.641 m/s	0.540 m/s	0.554 m/s	6.878 Pa	7.088 Pa	7.558 Pa
M7	1,781,979	0.625 m/s	0.531 m/s	0.559 m/s	6.975 Pa	7.199 Pa	7.641 Pa
Difference	M1-M2	2.184%	3.974%	2.232%	9.338%	4.847%	5.954%
	M2-M3	2.454%	0.579%	0.671%	0.854%	6.230%	7.163%
	M3-M4	0.306%	0.135%	1.030%	9.706%	0.619%	5.173%
	M4-M5	1.859%	0.513%	0.811%	0.662%	1.191%	1.675%
	M5-M6	2.379%	1.583%	0.306%	3.389%	1.768%	3.014%
	M6-M7	2.555%	1.776%	0.976%	1.393%	1.53%	1.086%

DOWN1, DOWN2 and DOWN3 represent three downstream planes distal to the valve; NCL, RCL and LCL stand for non-coronary, right coronary and left coronary leaflet, respectively

Computational results for flow characteristics and valve motion were analysed at representative time points throughout the third cardiac cycle. All postprocessing was conducted in FlowVision Viewer 3.13.04 (CAPVIDIA, Leuven, Belgium), Abaqus Visualization module 2019 (SIMULIA, Dassault System, France), EnSight 2020 R2 (Ansys Inc, USA), ParaView 5.9.1 (Kitware, New York, USA), and MATLAB R2022b (The MathWorks, MA, USA). WSS is an important haemodynamic parameter; its magnitude and directional variation have been correlated with aortic diseases and valve degeneration (Salmasi et al. 2021; Hanna et al. 2022). Both instantaneous and time-averaged wall shear stress (TAWSS) on the aortic wall and leaflets were analysed. Other time-averaged metrics, including oscillatory shear index (OSI) and relative residence time (RRT), were also evaluated and compared among the three leaflets. Their definitions are given below:

2 $${\text{TAWSS}} = { }\frac{{\mathop \smallint \nolimits_{0}^{T} \left| {{\varvec{\uptau}}} \right|{\text{dt}}}}{T}$$

3 $${\text{OSI}} = { }\frac{1}{2}\left( {1 - \frac{{\left| {\mathop \smallint \nolimits_{0}^{T} {{\varvec{\uptau}}}{\text{dt}}} \right|}}{{\mathop \smallint \nolimits_{0}^{T} \left| {{\varvec{\uptau}}} \right|{\text{dt}}}}} \right)$$

4 $${\text{RRT}} = { }\frac{1}{{\left( {1 - 2 \times {\text{OSI}}} \right) \times {\text{TAWSS}}}}$$

where ${\varvec{\uptau}}$ represents the instantaneous WSS vector, and *T* is the cardiac cycle.

## Results

### Flow patterns

The key haemodynamic parameters are summarized and compared among the four simulation cases and with the 4D-flow MRI measurements in Table 5. Stroke volume (SV) was calculated by integrating flow rate over time during the systolic phase. The maximum jet velocity and peak systolic spatial mean velocity were measured at 15 mm distal to the sinus central plane. It can be observed that implementing the non-Newtonian fluid property and incorporating the coronary arteries have a minor influence (less than 6%) on these key haemodynamic parameters: stroke volume varied by 1.4% (123.52 to 126.10 mL), maximum jet velocity by 4.4% (2.264 to 2.367 m/s), and peak systolic spatial mean velocity by 5.3% (1.012 to 1.066 m/s). It is also clear that all four models can predict the key haemodynamic parameters accurately, with errors of up to 8% for SV, 5% for the maximum jet velocity, and 2% for peak systolic spatial mean velocity when compared to the corresponding in vivo measurements.

**Table 5:** Quantitative comparison of key haemodynamic parameters derived from simulation results for the four cases and with the corresponding MRI measurements

Parameter	NC-N	C-N	NC-NN	C-NN	MRI
Stroke volume (mL)	124.24	124.06	123.52	126.10	134.32
Maximum jet velocity (m/s)	2.367	2.285	2.264	2.307	2.388
Peak systolic spatial mean velocity (m/s)	1.043	1.066	1.026	1.012	1.041

**Fig. 3:**
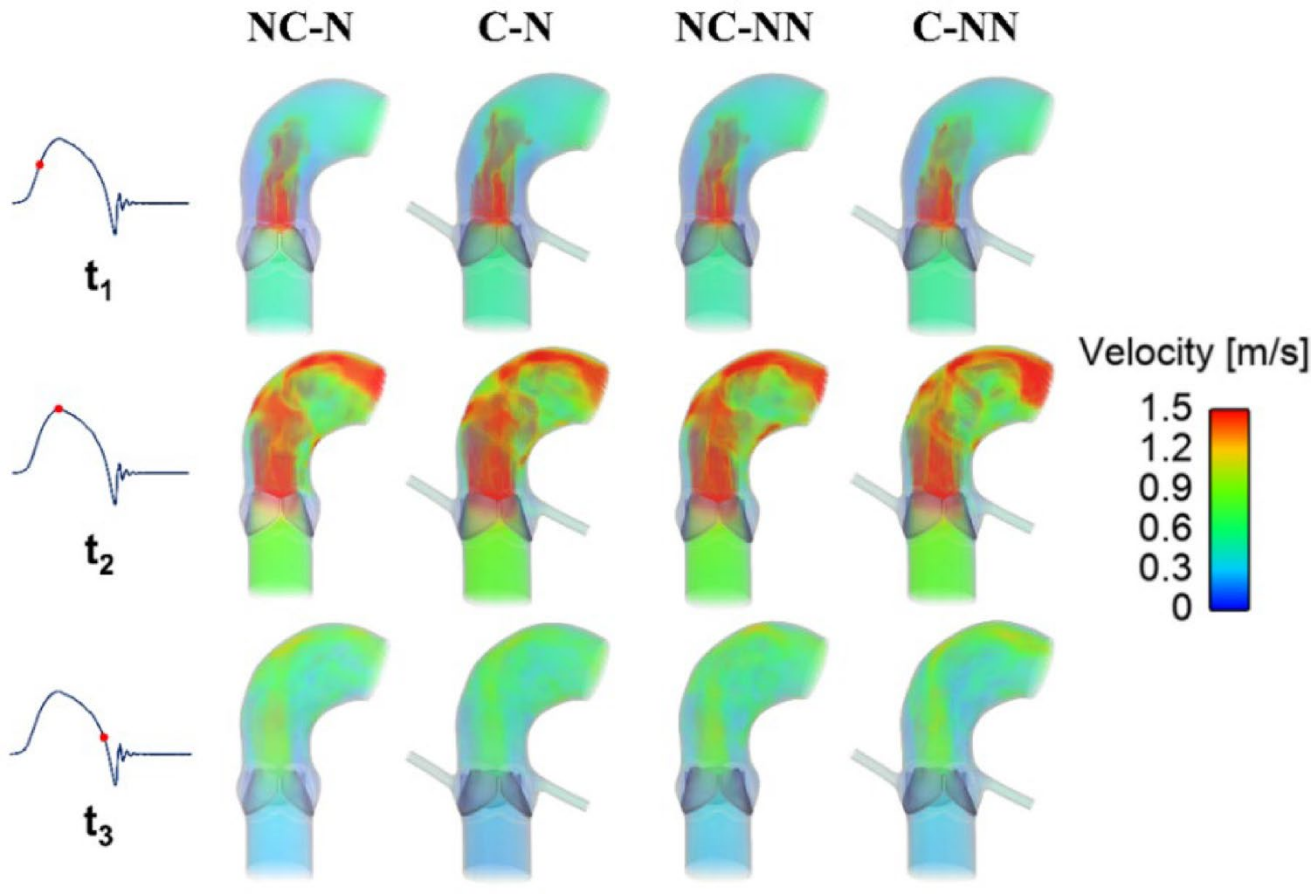
Velocity 3D contours at three representative time points (t_1_: acceleration phase, t_2_: peak systole, and t_3_: deceleration phase) for the four simulated cases

Figure 3 shows velocity 3D volume rendering contours for the four simulated cases. Overall, the general spatial distribution is very similar in the area upstream of the valve. At the acceleration phase (t_1_), all cases see a high-velocity jet emerging through the opening valve. At peak systole (t_2_), high-velocity flow has reached the arch and is directed towards the descending aorta. At the deceleration phase (t_3_), the flow jet striking the outer curvature of the aortic wall shows a noticeable reduction in velocity compared to the previous phase, while in the region between this flow and the inner curvature, the cases with coronary arteries (C-N and C-NN) exhibit a slightly lower velocity magnitude.

**Fig. 4:**
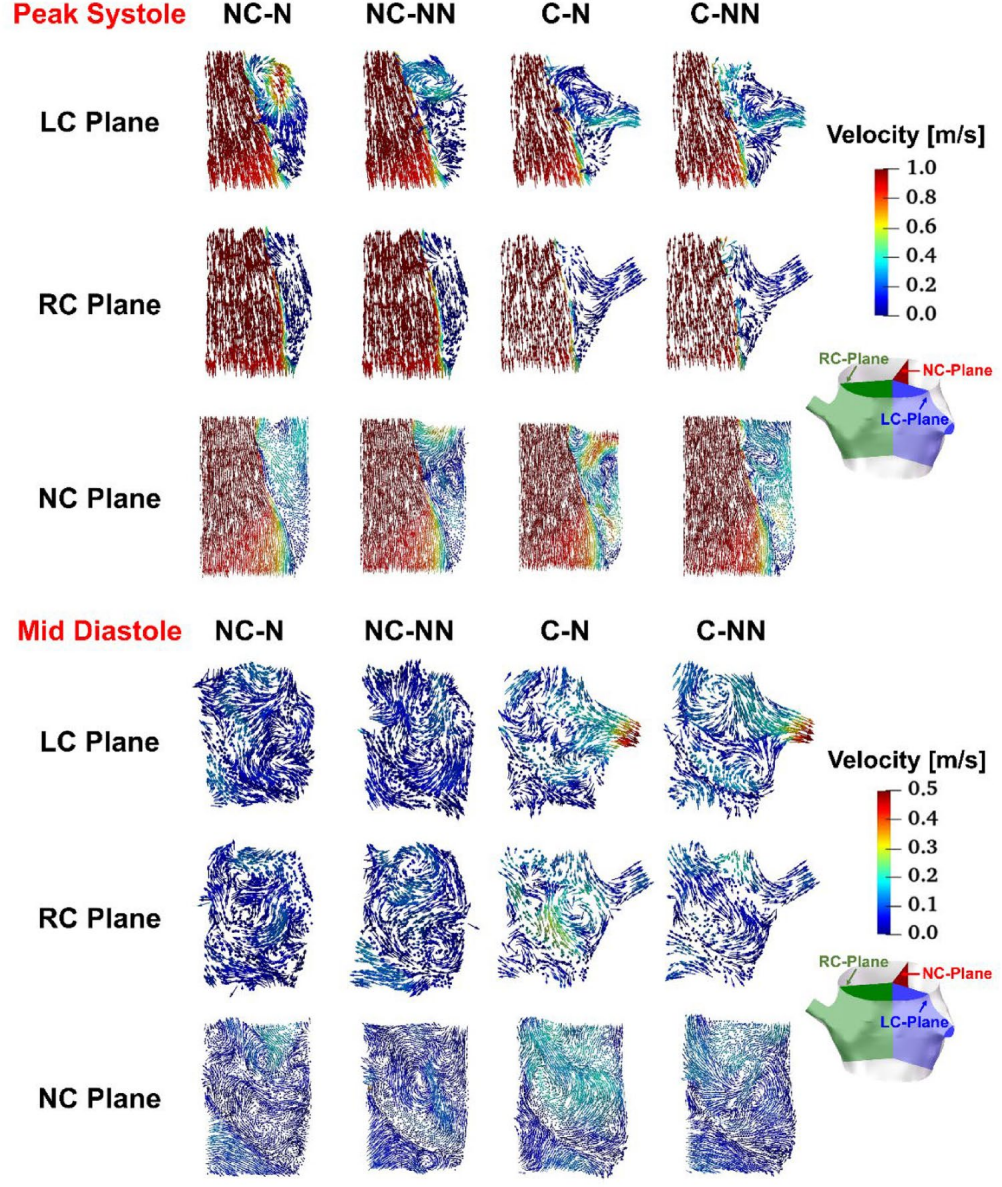
Velocity vectors on the bisecting plane of LC, RC, and NC sinus at peak systole and mid-diastole for the four simulated cases. *LC* left coronary, *RC* right coronary, *NC* non-coronary

Figure 4 shows velocity vector plots on the bisecting plane of each sinus for the four simulated cases at peak systole and mid-diastole. The impact of coronary outflow can be visualized clearly. The inclusion of coronary arteries causes a substantial portion of flow near the sinus wall to be diverted to the coronary ostium rather than being recirculated towards the free stream jet in the left and right coronary sinuses, which can be seen at both time points. When non-Newtonian fluid properties are included, there is some reduction in velocity in the space between the leaflet and the sinus wall.

### Valve dynamics

**Fig. 5:**
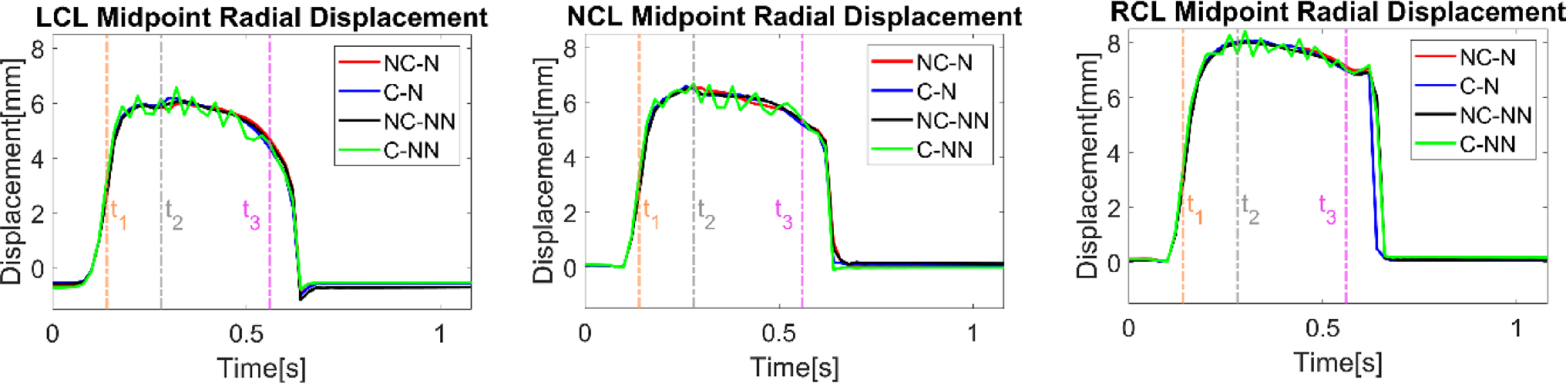
Variations of midpoint displacements for LCL, NCL, and RCL throughout the cardiac cycle for the four simulated cases. t_1_: acceleration phase, t_2_: peak systole, and t_3_: deceleration phase

Figure 5 shows variations of midpoint displacements for LCL, NCL, and RCL of the valve during a cardiac cycle. For all three leaflets, their midpoint radial displacements with respect to the initial unloaded positions reach a maximum at peak systole. The four cases show a similar trend in valve dynamics except for C-NN, which displays oscillations around the maximum displacement until the valve is closed. The amplitudes of oscillations are similar for the three leaflets, with the frequency gradually decreasing over time.

**Fig. 6:**
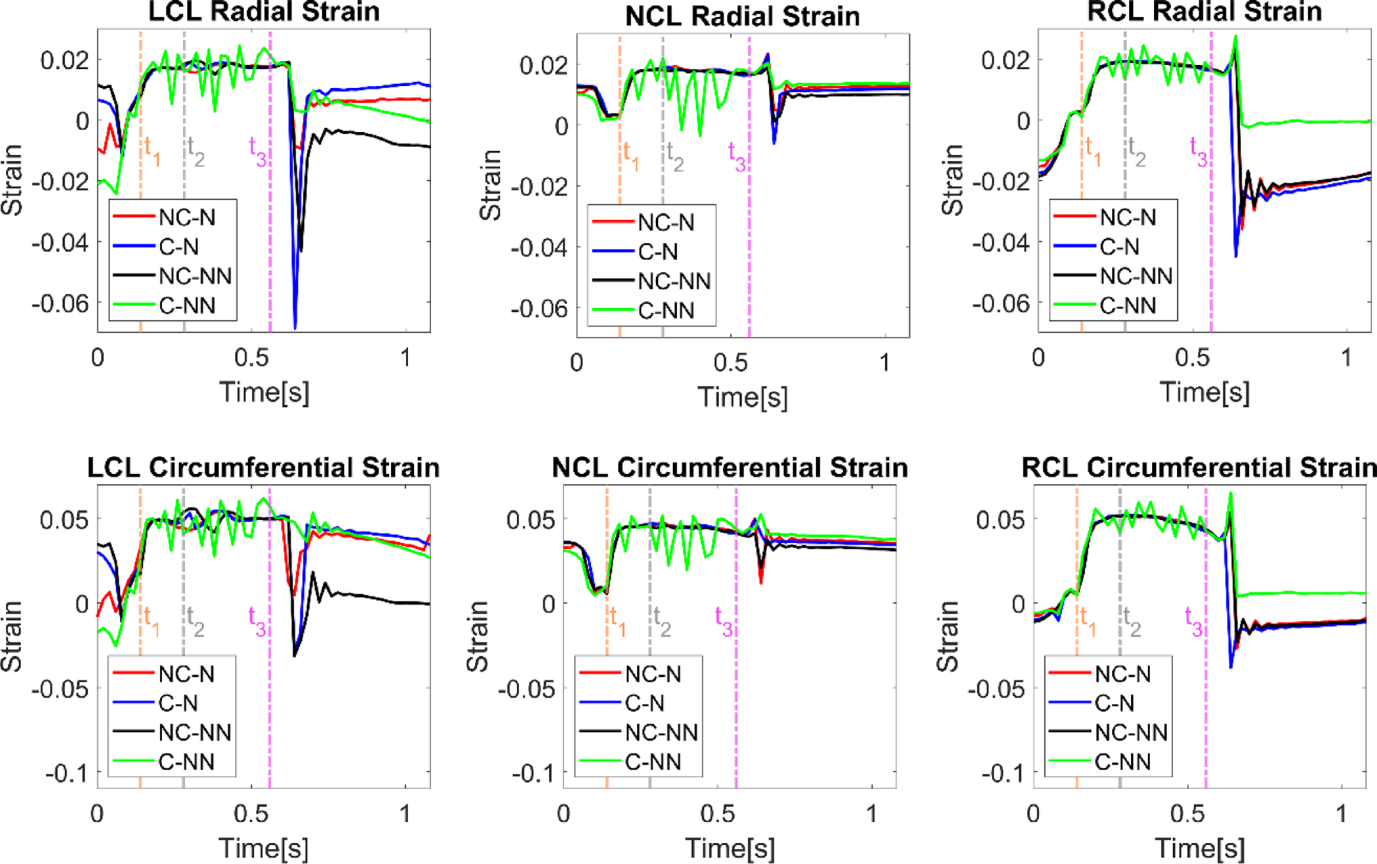
Variations of leaflet radial and circumferential strains throughout the cardiac cycle for the four simulated cases. t_1_: acceleration phase, t_2_: peak systole, and t_3_: deceleration phase

Figure 6 shows variations of the radial and circumferential strains for each leaflet during a cardiac cycle. Generally, the circumferential strains are greater than the radial strains, and both exhibit a similar time-varying trend for each leaflet. Among the three leaflets, the RCL and LCL display more pronounced variations over time compared to the NCL. All four cases follow a similar trend during most of the cardiac cycle, except for C-NN, which exhibits intense oscillations during systole. The C-NN case also shows near-zero strains in the RCL during diastole, while the other three cases have negative strains in the RCL.

### Spatial distribution of viscosity for the non-Newtonian cases

**Fig. 7:**
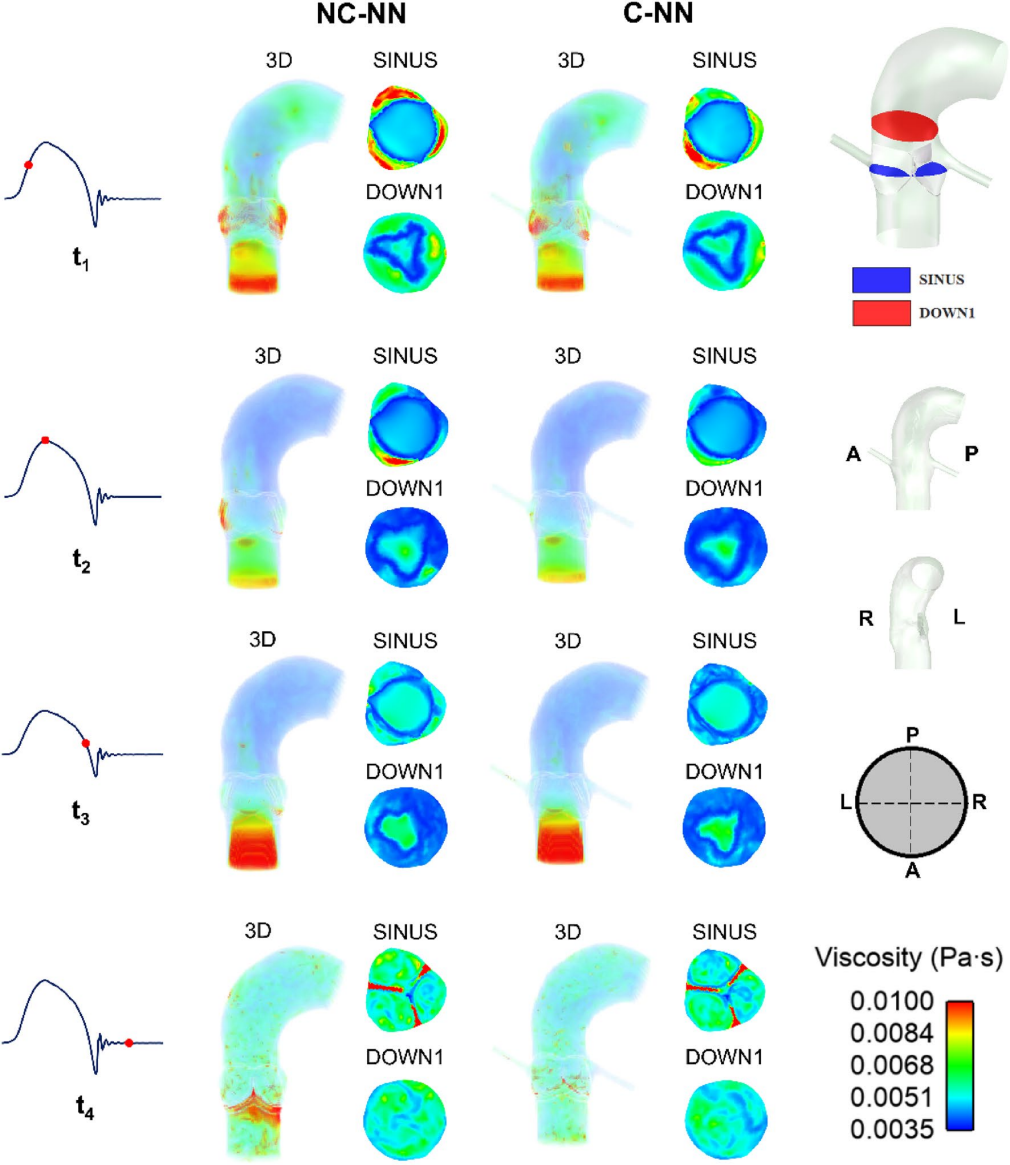
Spatial viscosity distributions (3D, SINUS and DOWN1 plane) at four representative time points (acceleration phase, peak systole phase, deceleration phase, and mid-diastolic phase) for the NC-NN case and C-NN case. A, P, R, and L represent anterior, posterior, right, and left direction, respectively

Spatial distributions of viscosity are shown in Fig. 7 for NC-NN and C-NN at four different time points. Viscosity distributions do not have a notable difference between the models with and without the coronary arteries in all areas except the sinus where flow is altered by of coronary outflow. In both cases, viscosity is highest in the sinus region during systolic acceleration when the valve is beginning to open. Inclusion of coronary arteries reduces the viscosity in the sinuses.

### Instantaneous wall shear stress

**Fig. 8:**
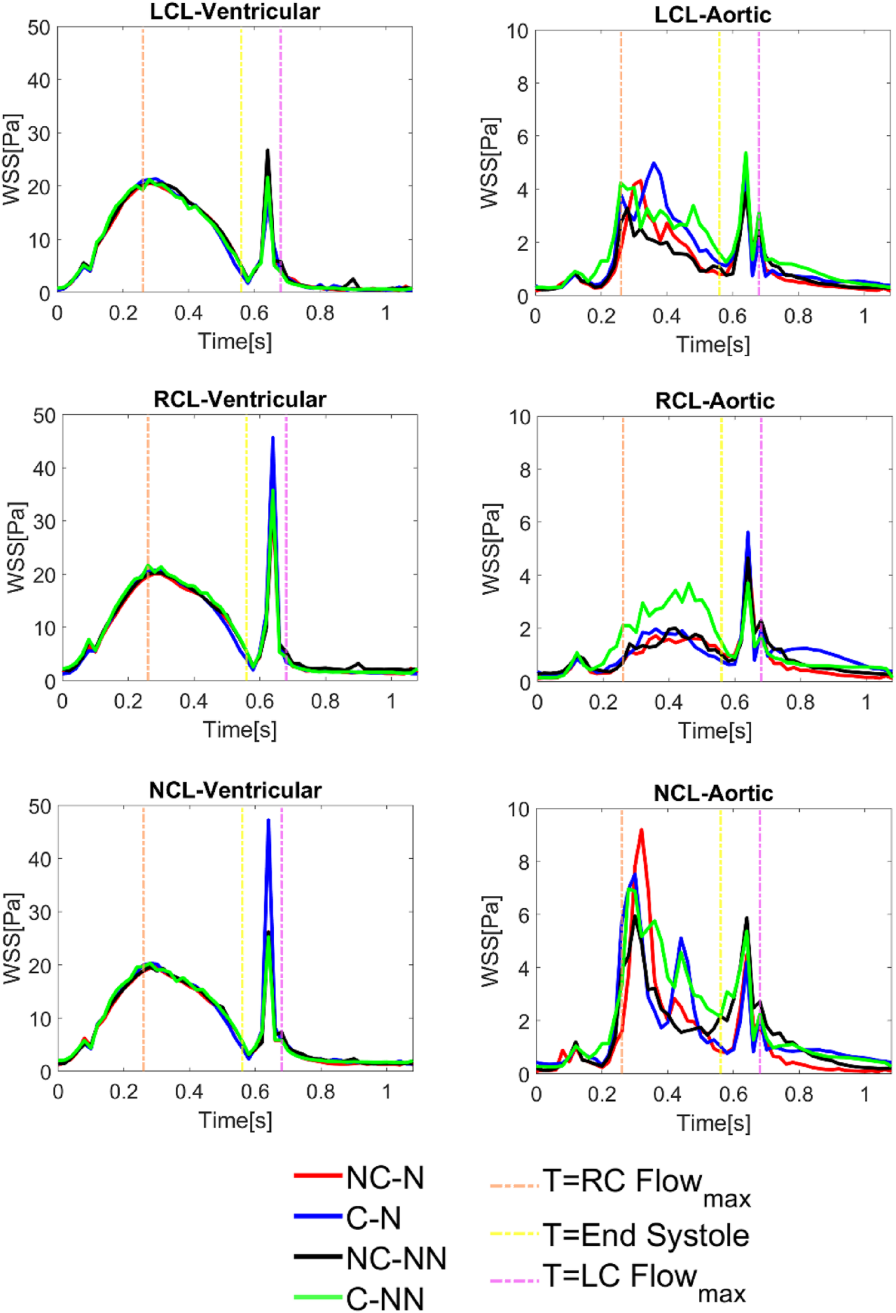
Instantaneous wall shear stress on both sides of the leaflets for the four simulated cases

Figure 8 shows the instantaneous wall shear stress on both sides of the leaflets for the four simulated cases. On the ventricular side, there is hardly any difference among the simulated cases and a general trend is that WSS reaches its maximum at peak systole, followed by a second peak at end-systole caused by the water hammer effect when the valve is closing. There are minimal differences in the maximum WSS magnitude during systole among the cases. On the aortic side, the effects of coronary outflow and non-Newtonian properties are more obvious. Models with coronary arteries (C-N, C-NN) have higher WSS on the LCL and RCL than those without (NC-N, NC-NN), while the opposite is true for the NCL. Including the non-Newtonian model has different effects on WSS on the three leaflets—it decreases WSS on the LCL and NCL but increases WSS on the RCL. Notably, the C-NN case has higher systolic WSS on the RCL compared to the other cases. Additionally, on the NCL aortic side, a mid-systole spike (at t = 0.44 s) is observed in both cases with coronary arteries. This phenomenon arises from the influence of coronary flow on local haemodynamics—as both the coronary flow and LVOT inflow decrease after peak systole, the coronary flow declines at a slower rate. Consequently, its proportion relative to the diminishing LVOT inflow becomes more significant, redistributing velocity near the NCL. This effect is absent in non-coronary cases, where sinus flow remains undisturbed by coronary outflow.

### Time-averaged wall shear stress

**Fig. 9:**
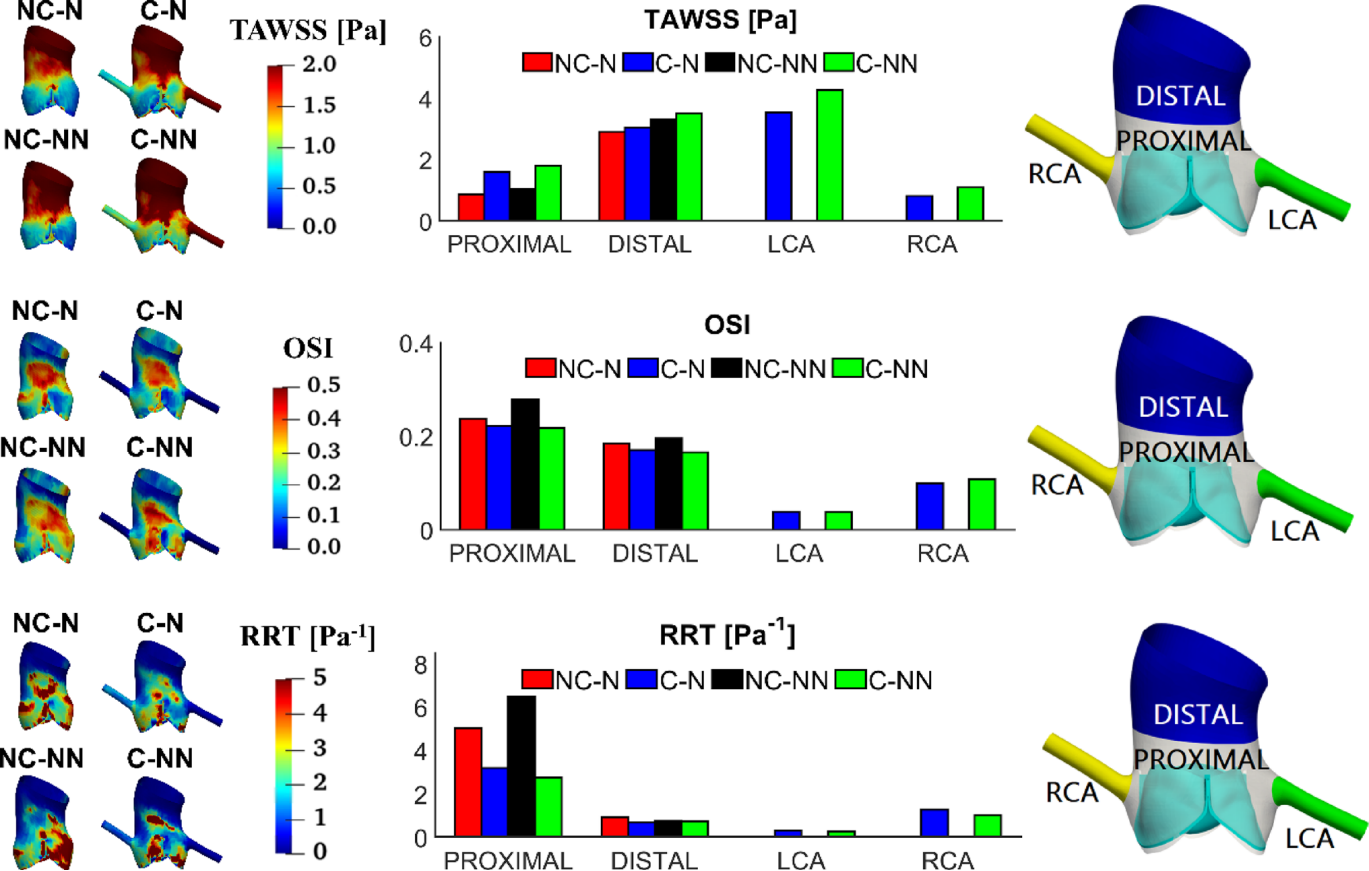
TAWSS, OSI and RRT in different areas of the aortic root and comparison of their spatially averaged values

Figure 9 shows the distribution of TAWSS, OSI, and RRT and their spatially averaged values in different areas of the aortic root. The overall trend is that the PROXIMAL region has lower TAWSS and higher OSI and RRT than the DISTAL region. Compared to the baseline case (NC-N), including the coronary arteries (C-N) leads to an increase in TAWSS and reductions in OSI and RRT in the aortic root, whereas accounting for the non-Newtonian behaviour in the absence of coronary arteries (NC-NN) causes OSI and RRT to increase, especially in the PROXIMAL region. When both coronary flow and non-Newtonian properties are incorporated (C-NN), higher TAWSS (by up to 108.45%), lower OSI (8.02%) and smaller RRT (44.62%) are obtained in the aortic sinuses (PROXIMAL region). Among the four simulated cases, the baseline model predicted the lowest TAWSS (0.87 Pa) in the aortic root, while its non-Newtonian version (NC-NN) produced the highest OSI (0.27) and RRT (6.47 Pa^−1^), all in the PROXIMAL area. It can also be observed that the influence of non-Newtonian behaviour is less significant when coronary outflows are included. Comparing the coronary arteries, the right coronary artery has lower TAWSS and higher OSI and RRT than the left coronary artery, and the non-Newtonian effect is trivial.

**Fig. 10:**
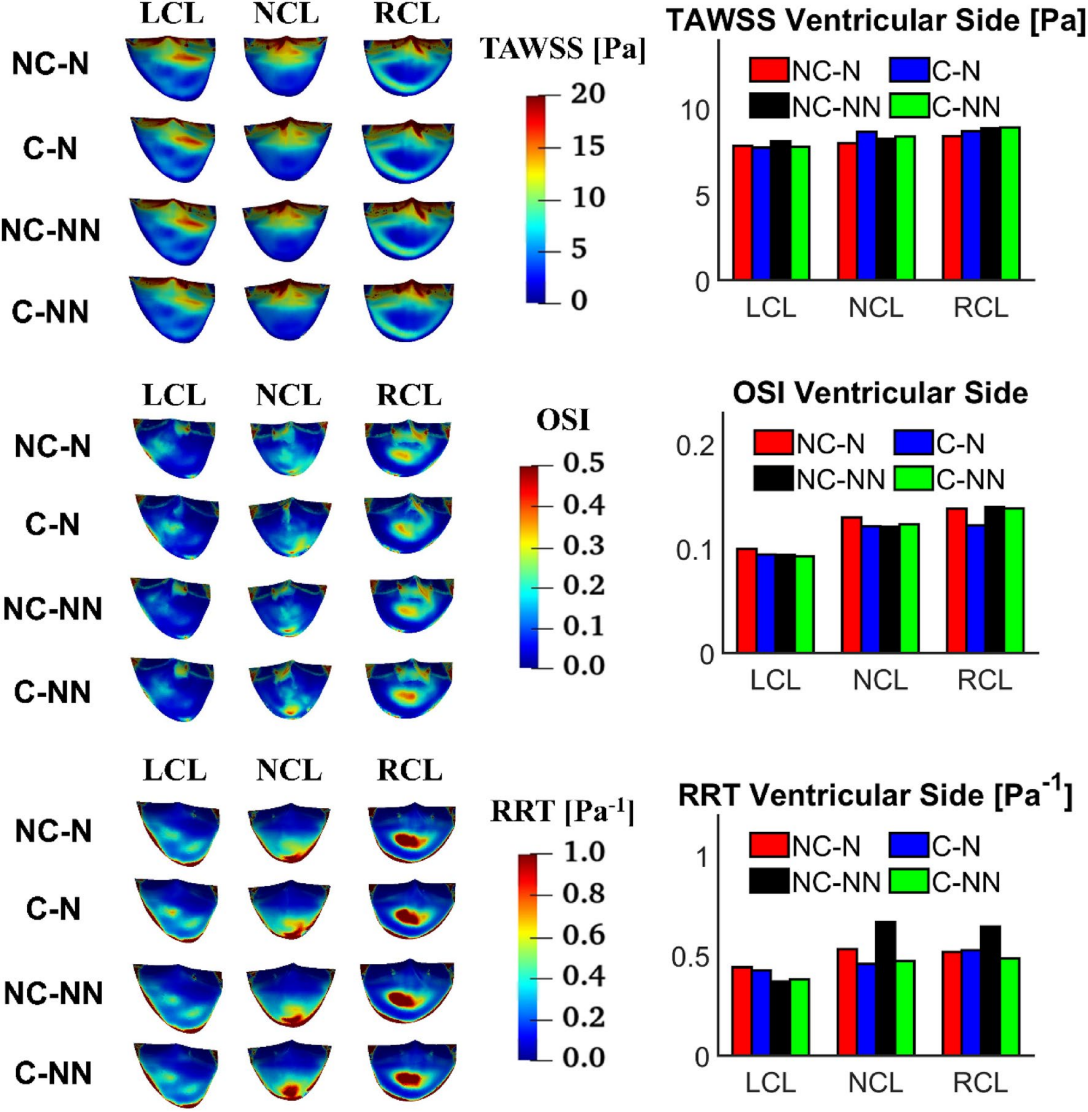
TAWSS, OSI and RRT on the ventricular side of the leaflets and comparison of spatially averaged values for each leaflet

Figure 10 shows the distributions of TAWSS, OSI and RRT on the ventricular side of the leaflets along with their spatially averaged values. The overall trend is that all leaflets have similar TAWSS magnitude on the ventricular side, and that accounting for coronary outflows and the non-Newtonian effect has little influence on the TAWSS values. OSI and RRT are more sensitive to the choice of model, but their values are not large enough to cause any concerns. Compared to the baseline case, the most notable difference is in RRT with NC-NN where RRT values on the ventricular side of NCL and RCL are increased.

**Fig. 11:**
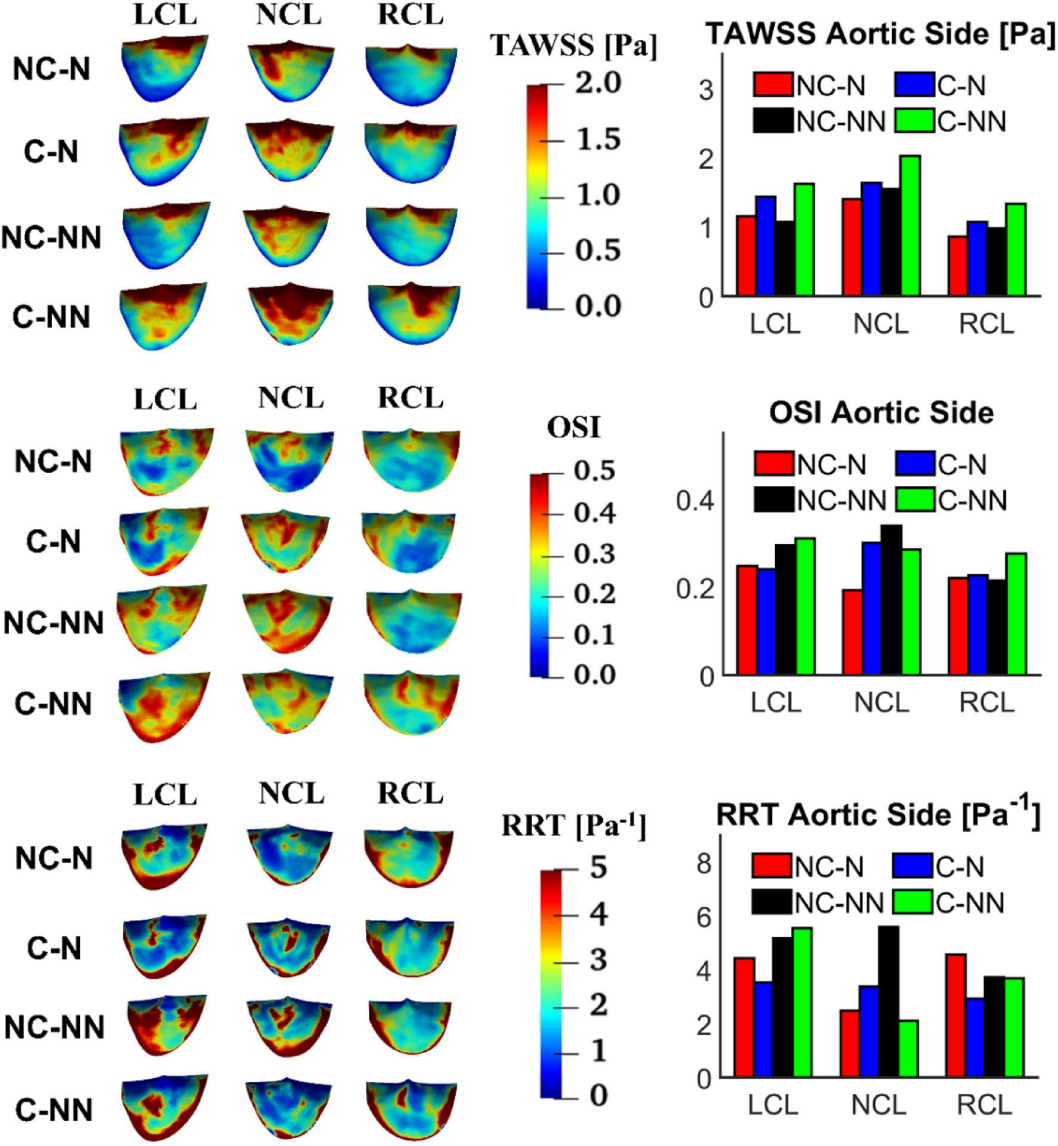
TAWSS, OSI and RRT on the aortic side of the leaflets and comparison of spatially averaged values for each leaflet

TAWSS, OSI and RRT on the aortic side of the leaflets are displayed in Fig. 11 along with their spatially averaged values. The overall trend is that TAWSS is lower on the RCL surface than LCL and NCL, while RRT is higher on LCL. Compared to the baseline case, incorporating the coronary arteries and non-Newtonian flow results in elevated TAWSS on all leaflets (41.04%, 44.76%, and 54.91% on the LCL, RCL and NCL, respectively), but its effect on OSI and RRT varies among the three leaflets. The most notable difference is in RRT with NC-NN where RRT values on the aortic side of NCL and RCL are increased. Compared to the ventricular side (Fig. 10), TAWSS on the aortic side is much lower, and OSI and RRT are higher for all leaflets.

## Discussion

The present study is an extension of our previous work on a healthy AoV (Yin et al. 2024) where the FSI simulation results were validated against in vivo 4D-flow MRI data. Since our previous FSI model did not include the coronary arteries and non-Newtonian viscosity, this study was designed to quantify their individual and combined effects on AoV dynamics and the surrounding haemodynamics. This was achieved by including the subject-specific coronary arteries in the aortic root model and employing the Bird–Carreau non-Newtonian model separately, followed by applying both changes simultaneously to the healthy AoV model. Two-way fully coupled FSI simulations were conducted with all other settings being kept consistent, except for the 3EWK parameters at the descending aorta outlet (Table 3), which differed slightly between models with and without the coronary arteries, to account for the different flow redistributions.

### Impact on flow pattern and valve dynamics

Compared to the baseline case (NC-N), incorporating the coronary arteries causes changes in local flow patterns resulting from outflows through the left and right coronary ostia in the aortic root. Figure 4 shows clearly how flow patterns in the aortic sinuses are altered as a proportion of flow finds its path through the coronary arteries, especially during diastole. Since coronary flows are a small fraction of the inlet flow, these changes are insignificant during systole. Nevertheless, the small adjustment in the outlet 3EWK parameters affects the transvalvular pressure difference during systole. Consequently, the key haemodynamic parameters also vary (Table 5), with the predicted SV differing by up to 2.05% between NC-NN and C-NN and the maximum jet velocity differing by up to 3.59% between NC-N and C-N.

**Fig. 12:**
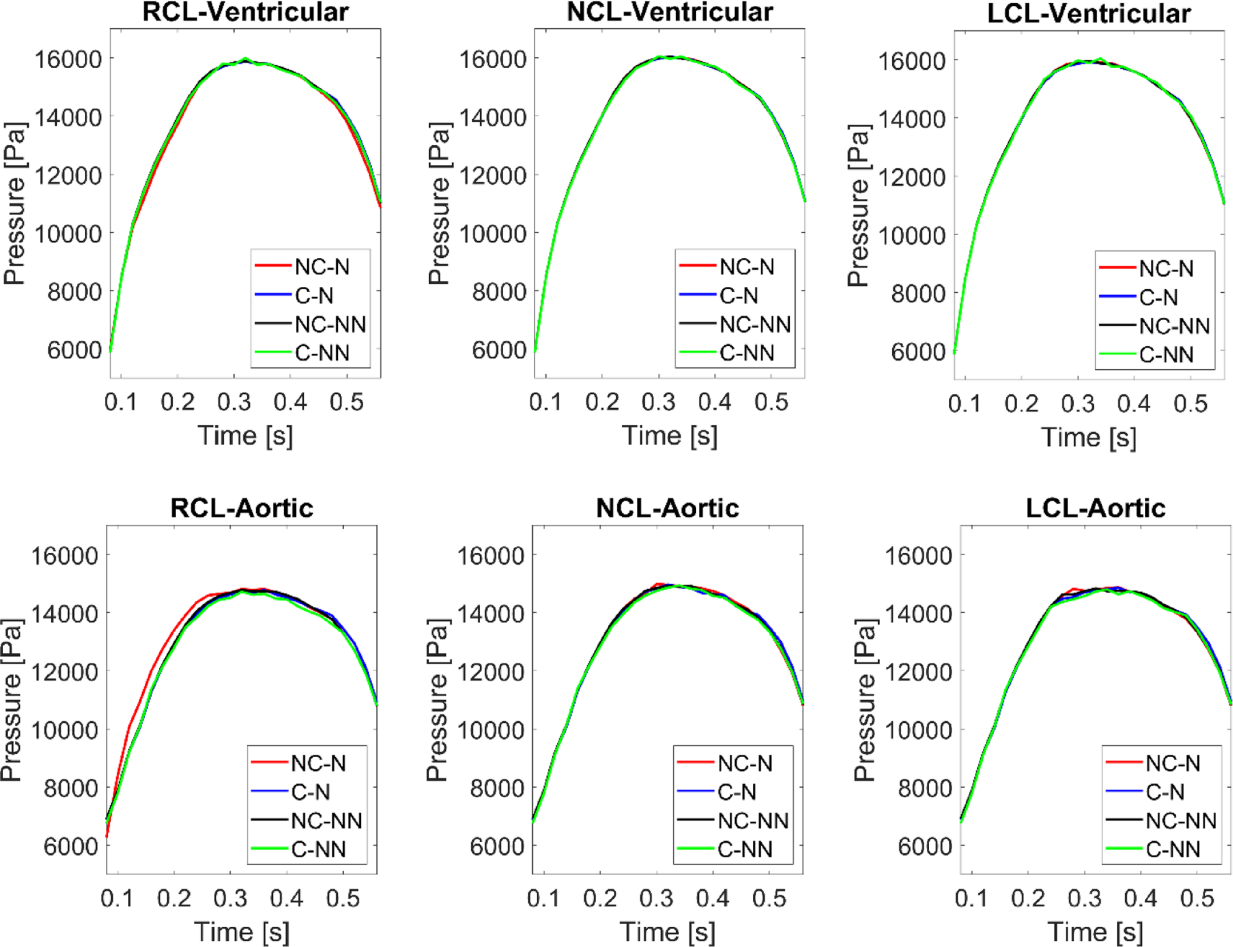
Pressure on the ventricular and aortic side of the three leaflets during systole for the four simulated cases

Accounting for the shear thinning non-Newtonian behaviour of blood results in increased viscosities in regions of relatively low velocity gradients, such as in the sinuses. Consequently, blood velocities in these areas decrease (Fig. 4). This reduction in flow velocity may promote the expansion of flow stagnation zones, thereby increasing the risk of thrombus formation. In spite of this, the non-Newtonian effect on flow characteristics and valve dynamics is minor in the absence of coronary outflows, which is consistent with previous findings (De Vita et al. 2015). However, when the coronary arteries are included, the fluid force on the adjacent leaflets starts to oscillate. Figure 12 shows the pressure on both sides of the three leaflets during systole, revealing that results in the C-NN case are more oscillatory, particularly on the ventricular side. This may be responsible for leaflet fluttering observed in this case (Figs. 5 and 6), but further investigation is required to understand the root cause of this behaviour.

**Fig. 13:**
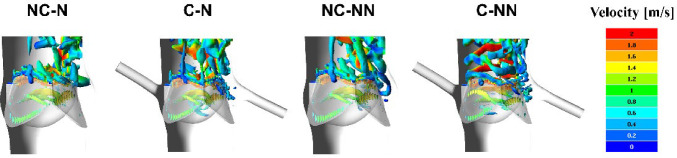
Visualisation of vortex cores surrounding the AV (using 3D isosurfaces of Q-criterion = 5 × 10^4^ s^−2^) at peak systole coloured by velocity magnitude

Additionally, vortical flow is observed in the C-NN case. Figure 13 shows the vortex cores at peak systole by using Q-criterion 3D isosurfaces of value 5 × 10^4^ s^−2^. A sequence of rotating vortex rings forms above the valve leaflets in the C-NN case, which is less evident in the other cases. The presence of vortices changes the local velocity gradient and shear stress, leading to nonuniform pressure which may cause leaflet fluttering. This highlights a potential interaction mechanism where coronary flow, in the presence of non-Newtonian effects, alters sinus vortex stability.

It is widely accepted that leaflet fluttering occurs in diseased valves (Sabbah et al. 1977) or artificial valves that are not perfectly designed (Avelar et al. 2017; Corso and Obrist 2024; Costa et al. 2024; Johnson et al. 2020). However, some studies using echocardiography showed that minor vibrations or fluttering could also be detected in normal valves without indicating pathology (Otto 2013; Bombardini et al. 2007; Lurie et al. 2003). This was thought to be caused by increased blood flow velocity, potentially during exercise or periods of increased cardiac output, leading to harmless flutter. Nevertheless, the observed vortex formation and leaflet fluttering in the C-NN case were derived from simulations under the rigid wall assumption, which is a serious limitation of the present study. Aortic root compliance is known to help ensure efficient valve leaflet closure (Flamini et al. 2016). Previous studies also demonstrated that accounting for aortic wall deformation could dampen flow oscillation and stabilise valve motion (Hsu et al. 2014; Marom et al. 2012). Consequently, the observed leaflet fluttering in the C-NN case should be re-evaluated in the future studies incorporating a compliant aortic wall.

### Impact on wall shear stress distribution on the aorta and leaflets

Including coronary outflow and non-Newtonian blood property can impact the distribution of WSS on the aorta and leaflets. Inclusion of coronary outflows leads to an increase in spatially averaged TAWSS in all areas of interest, except for the LCL on the ventricular surface. This finding aligns with previous observations in idealized models of the aorta with symmetric leaflets (Cao and Sucosky 2017; Moore and Dasi 2015). Incorporating the non-Newtonian blood property also results in elevated TAWSS through increasing viscosity throughout the aortic root. High TAWSS can increase the risk of endothelial damage, leading to aorta-pathology and valve degeneration (Salmasi et al. 2021; Hanna et al. 2022). It can also trigger platelet activation and promote thrombus formation.

It has been suggested that oscillatory shear and prolonged residence time are associated with intravascular abnormalities and thrombus formation (Mutlu et al. 2023; Laha et al. 2024); hence, it is important to evaluate OSI and RRT, especially on the aortic side of the leaflets. Although these parameters have been evaluated in recently published FSI studies of the aortic valve, (Fringand et al. 2024; Govindarajan et al. 2024; Laha et al. 2024; Qin et al. 2024; Xie et al. 2024), they either employed a Newtonian fluid assumption or omitted the coronary arteries. Our results show that applying the Newtonian fluid assumption and exclusion of coronary outflows could underestimate OSI and RRT on the aortic side of LCL and RCL (Fig. 11). This is attributed to the underestimation of blood viscosity and the washout effect of the coronary arteries (Moore and Dasi 2015). For the NCL, the velocity magnitude is lower in the area between the NCL and the sinus with the Newtonian assumption, resulting in higher OSI and RRT. These findings are associated with the asymmetric leaflet geometries employed in the current work and have not been reported in previous studies employing symmetric geometry (Cao and Sucosky 2017; Moore and Dasi 2015).

Overall, the effects of including the coronary outflow and non-Newtonian fluid property are greater on WSS and its related metrics (particularly on the aortic side of leaflets) than on flow patterns. Notably, coronary outflows play a more important role. Therefore, these two factors should not be overlooked when accurate predictions of WSS-related parameters are important, e.g. when assessing potential risks for thrombus formation.

### Limitations

This study involves several assumptions. First, the most serious assumption was the rigid aortic root, which is known to affect valve closure dynamics and may cause overestimation of WSS values. Secondly, the employed coronary flow waveforms were not subject-specific but scaled based on literature data, which could influence the accuracy of WSS predictions in the aortic sinuses and on the aortic side of the leaflets. Thirdly, the valvular flow may become turbulent during part of the cardiac cycle; hence, a laminar flow assumption is insufficient and the effect of turbulence should be included for more accurate predictions of flow patterns and vortex structure in the aortic sinuses (Becsek et al. 2020; Hellmeier et al. 2020; Mutlu et al. 2021). Finally, the study is limited to one healthy natural valve. The quantitative effects of non-Newtonian fluid and coronary outflow on the evaluated WSS parameters can vary with the aortic root and leaflet geometry. Future research should extend the FSI methodology to more healthy and coronary disease cases to explore any potential relationships between AoV function and coronary artery lesions.

## Conclusion

In this study, the individual and combined effects of coronary and non-Newtonian flow were examined by performing FSI analyses on a natural, healthy AoV. Results obtained from the four model combinations demonstrate that the influence of coronary flow is more pronounced than employing a non-Newtonian model, and their combined effect is non-negligible, particularly on WSS-related parameters. Compared to the baseline model (NC-N), incorporating coronary and non-Newtonian flow (C-NN) led to higher TAWSS in the aortic sinuses (by up to 108.45%), lower OSI (8.02%) and smaller RRT (44.62%). On the aortic side of valve leaflets, using the C-NN model increased TAWSS by 41.04%, 44.76%, and 54.91% on the left, right and non-coronary leaflet, respectively. These results highlight the importance of incorporating coronary outflow and non-Newtonian properties when accurate predictions of WSS-related parameters are critical.

## Data Availability

Data are provided within the manuscript.
